# 30-Day Mortality Following Palliative Radiotherapy

**DOI:** 10.3389/fonc.2021.668481

**Published:** 2021-04-23

**Authors:** Miriam Vázquez, Manuel Altabas, Diana C. Moreno, Abraham A. Geng, Santiago Pérez-Hoyos, Jordi Giralt

**Affiliations:** ^1^ Department of Radiation Oncology, Vall d’Hebron University Hospital, Vall d’Hebron Institute of Oncology, Barcelona, Spain; ^2^ Unit of Statistics and Bioinformatics, Vall d'Hebron University Hospital, Barcelona, Spain

**Keywords:** 30-day mortality, palliative radiation, end-of-life, prognosis, clinical indicator

## Abstract

**Purpose:**

30-day mortality (30-DM) is a parameter with widespread use as an indicator of avoidance of harm used in medicine. Our objective is to determine the 30-DM followed by palliative radiation therapy (RT) in our department and to identify potential prognosis factors.

**Material/Methods:**

We conducted a retrospective cohort study including patients treated with palliative RT in our center during 2018 and 2019. Data related to clinical and treatment characteristics were collected.

**Results:**

We treated 708 patients to whom 992 palliative irradiations were delivered. The most frequent primary tumor sites were lung (31%), breast (14.8%), and gastrointestinal (14.8%). Bone was the predominant location of the treatment (56%), and the use of single doses was the preferred treatment schedule (34.4%). The 30-DM was 17.5%. For those who died in the first month the median survival was 17 days. Factors with a significant impact on 30-DM were: male gender (p < 0.0001); Eastern Cooperative Oncology Group (ECOG) Performance Status (PS) of 2–3 (p = 0.0001); visceral metastases (p = 0.0353); lung, gastrointestinal or urinary tract primary tumors (p = 0.016); and single dose RT (p = <0.0001). In the multivariate analysis, male gender, ECOG PS 2–3, gastrointestinal and lung cancer were found to be independent factors related to 30-DM.

**Conclusion:**

Our 30-DM is similar to previous studies. We have found four clinical factors related to 30-DM of which ECOG was the most strongly associated. This data may help to identify terminally ill patients with poor prognosis in order to avoid unnecessary treatments.

## Introduction

Radiation therapy (RT) has a well-established role in the palliative approach of patients with cancer. When using palliative RT, symptom relief is usually obtained in a wide range of time which varies depending on the primary tumor, the location of the treatment or the patient’s health. However, when survival is too short, these patients may die before they benefit from RT.

The use of chemotherapy in dying patients has been previously reported as an aggressive and poor tailored end-of-life care indicator (1). In the last years the use of RT at the end of life has also been a matter of concern (2–4). When proposing a palliative treatment with radiation, the presumed survival is an essential factor taken into account and may condition fractionation regimen. Therefore, the use of a larger number of fractions in terminally ill patients is likely to require spending a significant amount of their final days visiting a radiation therapy suite (2, 3). This has been suggested to be a consequence of an overoptimistism at survival prediction of dying patients (5).

The National Health Service of the United Kingdom proposed the 30-day mortality (30-DM) parameter as an indicator of aggressive management at the end of the life. Thus, when the estimated survival is less than one month, palliative RT is unlikely to be beneficial. The Royal College of Radiologist agreed that less than 20% of patients receiving palliative RT should die within 30 days of treatment (6). Therefore, it’s important to identify these patients with shortened survival and to carefully consider if the treatment should be avoided or not.

The use of palliative RT in the last 30 days of life varies substantially between centers and ranges between 0.7 and 33% (7). The purpose of this study was to determine 30-DM in patients who have received palliative RT in our center and to identify potential prognostic factors for 30-DM.

## Material and Methods

We performed a retrospective study including adult patients treated with palliative RT in our center from January 2018 to December 2019. Exclusion criteria were: patients under the age of 18, hematologic tumors, non-melanomatous skin cancer, treatment with stereotactic body radiation therapy or radiosurgery, and when survival status at 30 days was unavailable. All RT treatments were identified using Aria^®^ (Varian Medical Systems) which is a specific electronic record for patients referred for RT. Clinical data was recorded from the hospital electronic medical record. The study was approved by the institutional ethics committee of our center.

Demographic data, radiation treatment parameters, and disease characteristics were collected for each patient. The type of primary tumor was classified into eight groups according to the most frequent tumors: lung, breast, prostate, gastrointestinal, urinary tract, gynecological, head and neck, and others. Episodes were identified when the treatment intent was registered as palliative by a radiation oncologist, and radiotherapy was delivered in less than 15 fractions. Site of the treatment was allocated by primary tumor, bone, brain, lymph nodes, and soft tissue. For patients who were treated more than once, we took into account the last treatment to avoid data duplication. All patients were followed for at least one month and until 6 months.

The primary endpoint of our study was to determine 30-DM. The secondary endpoint was to identify potential prognostic factors in our cohort. 30-DM was assessed from the start of treatment to the moment of death. Patients were grouped according to their vital status within 30 days from the start of treatment: group “better survival” (BS) for survivors and group “lower survival” (LS) for non-survivors at 30 days. All patients were followed up during that period and none was lost. A descriptive analysis was carried using Chi-squared or Exact Fisher as adequate. Potential clinical and dosimetric variables related to mortality were checked fitting a univariate and multivariate logistic regression. Variables that improve the likelihood (p < 0.1) were included in the final mode. Covariates considered were the following: age, sex, ECOG PS, primary tumor, presence of visceral disease, treatment location, number of fractions, and reirradiation. A multivariable analysis was performed to identify independent prognostic factors. A Kaplan–Meier survival curve with six months of follow-up has been also estimated. All analyses were carried out with Stata 15.1.

## Results

**Figure 1:**
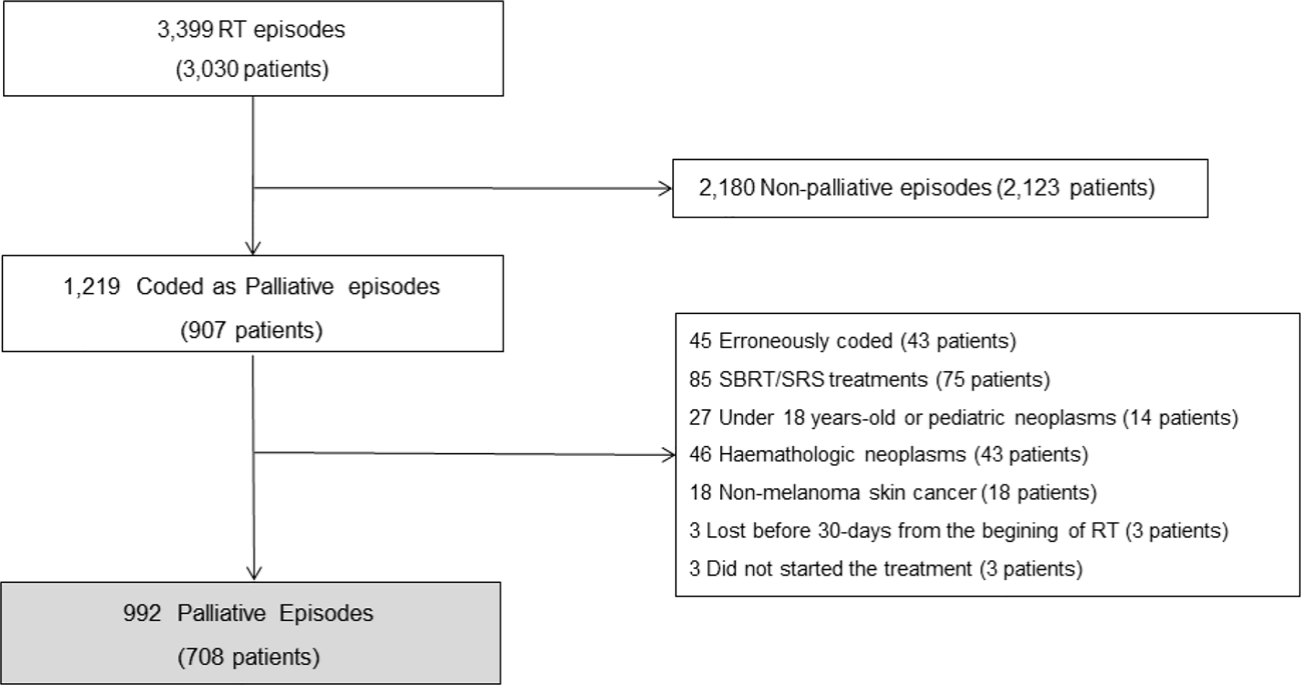
Consort diagram demonstrating exclusions from the study population.

A total of 708 patients were analyzed. **Figure 1** shows the consort flow diagram. The median age of the entire population at treatment was 66 years, male gender was predominant (58.2%), and the majority had a good performance status with an ECOG PS of 0–1 (59%). The most prevalent tumors were lung, breast, and gastrointestinal (31, 14.8, and 14.8% respectively). Bone was the most frequent site of radiation (56%), and the preferred schedule was single doses (34.4%) followed closely by 10–15 fractions (34%). The completion rate of the treatment was 94.8%. No differences were found according to age, location of the treatment, and reirradiation between groups. Of the 37 patients who did not end the treatment, 28 belonged to the LS group. Patient’s characteristics are shown in **Table 1**.

**Table 1:** Sample characteristics and descriptive analysis according to the state at 30-days.

	All patients(n = 708)	BS Group(n = 584)	LS Group(n = 124)	*p* value
**Age**				0.9865
18–64 years	327 (46.2%)	267 (45.7%)	60 (48.4%)	
≥ 65 years	381 (53.8%)	317 (54.3%)	64 (51.6%)	
**Sex**				**0.0001**
Male	412 (58.2%)	320 (54.8%)	92 (74.2%)	
Female	296 (41.8%)	264 (45.2%)	32 (25.8%)	
**ECOG PS**				**<0.0001**
ECOG PS 0-1	418 (59%)	380 (65.1%)	38 (30.6%)	
ECOG PS 2-3	290 (41%)	204 (34.9%)	86 (69.4%)	
**Primary tumor**				**0.0016**
Breast	105 (14.8%)	100 (17.1%)	5 (4%)	
Gastrointestinal	105 (14.8%)	79 (13.5%)	26 (21%)	
Lung	219 (31%)	169 (28.9%)	50 (40.3%)	
Prostate	82 (11.6%)	70 (12%)	12 (9.7%)	
Urinary tract	72 (10.2%)	59 (10.1%)	13 (10.5%)	
Gynecological	40 (5.6%)	37 (6.3%)	3 (2.4%)	
Head and neck	34 (4.8%)	28 (4.8%)	6 (4.8%)	
Other	51 (7.2%)	42 (7.2%)	9 (7.3%)	
**Visceral metastases**				**0.0353**
Present	414 (58.5%)	331 (56.7%)	83 (66.9%)	
Absent	294 (41.5%)	253 (43.3%)	41 (33.1%)	
**Location of the treatment**				0.6162
Bone	397 (56%)	324 (55.5%)	73 (58.9%)	
Brain	181 (25.6%)	153 (26.2%)	28 (22.6%)	
Pimary tumour	70 (9.9%)	56 (9.6%)	14 (11.3%)	
Soft tissue	43 (6.1%)	35 (6%)	8 (6.5%)	
Lymph nodes	17 (2.4%)	16 (2.7%)	1 (0.8%)	
**Number of fractions**				**<0.0001**
Single dose	243 (34.4%)	187 (32%)	56 (45.2%)	
2-9 fractions	224 (31.6%)	171 (29.3%)	53 (42.7%)	
10-15 fractions	241 (34%)	226 (38.7%)	15 (12.1%)	
**Reirradiation**				0.6242
Yes	66 (9.3%)	53 (9.1%)	13 (10.5%)	
No	642 (90.7%)	531 (90.9%)	111 (89.5%)	
**End of the treatment**				0.6242
Yes	671 (94.8%)	575 (98.5%)	96 (77.4%)	
No	37 (5.2%)	9 (1.5%)	28 (22.6%)	

Bold values: statistically significant.

**Figure 2:**
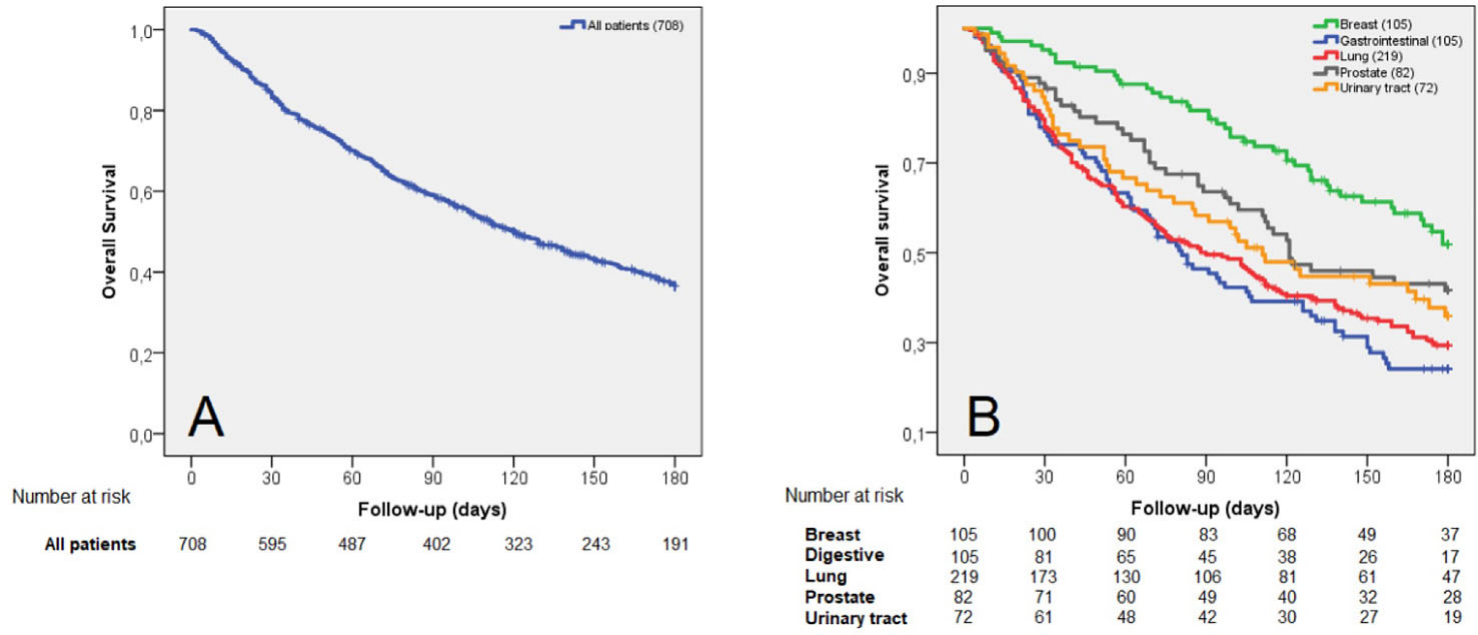
Survival curves including: all patients **(A)** and the five more prevalent primary tumors **(B)**.

Overall, 124 out of 708 patients died at 30 days (17.5%). The median survival was 17 days for the LS group. For the entire cohort, the median survival was 120 days (**Figure 2A**). Descriptive analysis according to the state at 30-days showed a higher prevalence in the LS group of ECOG 2–3 (p = 0.0001), male gender (p < 0.0001), visceral metastasis (p = 0.0353), and use of single doses (p < 0.0001). Primary tumor distribution between groups was different (p = 0.016) with a higher prevalence of lung, gastrointestinal, urinary tract, and other tumors in the LS group. Survival according to primary tumor is shown in **Figure 2B**.

The multifactorial analysis shows that male patients were 58% more likely to die within the first month after RT in comparison to female patients (OR 2.37, 95% CI: 1.5; 3.66). ECOG PS was the parameter with the highest impact in 30-DM with an increased risk of 77% of dying for those with an ECOG PS 2–3 (OR 4.22, 95% CI: 2.78; 6.40). According to primary tumor, lung and gastrointestinal neoplasms were also related to 30-DM (OR 1.66, 95% CI: 1.11; 2.48 and OR 1.7, 95% CI: 1.04; 2.78). In patients in whom visceral metastases were present, an increased risk of dying in the first month of 36% was assessed (OR 1.55, 95% CI: 1.03; 2.33). Age, treatment site, and reirradiation did not show any impact in 30-DM (**Table 2**). After adjusting for other characteristics, the multivariate analysis found that male sex, ECOG PS 2–3, gastrointestinal and lung tumors were found to be independent related factors to 30-DM. Although visceral metastases confidence interval includes 1, a trend toward a higher mortality was observed (OR 1.53, 95% CI: 0.98; 2.40) (**Table 3**).

**Table 2:** Univariate analysis investigating potential risk factors of 30-DM.

	OR	CI 95%	*p value*
**Age**			0.8460
18–64 years	1	1	
≥ 65 years	1.001	(0.987; 1.016)	
**Sex**			**0.0001**
Female	1		
Male	2.37	(1.5.; 3.66)	
**ECOG PS**			**<0.0001**
ECOG PS 0–1	1		
ECOG PS 2–3	4.22	(2.78; 6.40)	
**Primary tumor**			
Breast	1		
Lung	1.66	(1.11; 2.48)	**0.0133**
Prostate	0.79	(0.41; 1.50)	0.4665
Gastrointestinal	1.7	(1.04; 2.78)	**0.0358**
Urinary tract	1.04	(0.55; 1.97)	0.8985
Gynecologic	0,367	(0,111; 1,208)	0,0990
Head and neck	1.01	(0.41; 2.49)	0.9833
Other	1.01	(0.48; 2.13)	0.9793
**Visceral Metastases**			
Absent	1		
Present	1.55	(1.03; 2.33)	**0.0362**
**Location of the treatment**			0.6517
Primary tumor	1		
Bone	0.90	(0.44; 1.71)	
Brain	0.73	(0.36; 1.49)	
Lymph nodes	0.25	(0.003; 2.05)	
Soft tissue	0.91	(0.35; 2.40)	

Bold values: statistically significant.

**Table 3:** Multivariate analysis investigating potential risk factors of 30-DM.

	OR	CI 95%	*p* value
**Sex**			
Female	1		
Male	2.38	(1.538; 3.703)	**0.0001**
**ECOG PS**			
ECOG PS 0-1	1		
ECOG PS 2-3	4.38	(2.84; 6.74)	**<0.0001**
**Primary tumor**			
Lung	1.66	(1.11; 2.48)	**0.0133**
Gastrointestinal	2.38	(1.32; 4.27)	**0.0038**
**Visceral metastases**			
Absent	1		
Present	1.53	(0.98; 2.40)	0.0606

Bold values: statistically significant.

## Discussion

30-DM after palliative RT observed in our center was 17.5% of the palliative treatments. For those who died in the first month, the median survival was 17 days. A recent systematic review, showed an overall use of palliative RT rates in the last 30 days of life of 9–15.3% (7). Our results are slightly higher than previous studies (2–4, 7–12), but they are still adjusted to The Royal College of Radiologist recommendation of 30-DM to be inferior to 20%. Therefore, we consider that the selection of our patients for palliative treatment is adequate.

Park and et al. (7) conducted a systematic review and found that major predictors for 30-DM among single institution studies were ECOG PS, lung cancer primary, bladder cancer primary, multiple metastases, and evidence of progressive disease. In our analysis, we have also found the presence of a gastrointestinal tumor to be associated with 30-DM. Our center receives many patients with multi-treated digestive tumors for phase I trials. We believe this fact may partly explain this data. We have not found the presence of visceral metastases to have a statistically significant impact on survival in the multivariate analysis, but there is a clear trend we cannot ignore.

Studies analyzing patients with bone metastases treated with RT show a rather wide range of 30-DM. Ellsworth et al. (13), reported a 30-DM of 26%. The most frequent scheme consisted of 6–10 fractions (56%), while the use of single doses was 8%. On the other hand, a large Canadian population cohort study including 8,301 patients with bone metastases, showed a 30-DM of 14.5%, and a single dose was used in 64.2% of the patients in the last month of life (14). This imbalance is thought to be multifactorial and partially related to historical practice patterns and financing of the treatments. When considering patients treated for bone metastases in our series, 30-DM was 18.3%. The use of single doses was by far the most used (91%). These results are adjusted to international recommendations of a use of single fractions in patients with advanced cancer who have uncomplicated bone metastases (15, 16). Although single fractions schemes seem to be advantageous in terminally ill patients, published data show that the use multiple fractions are preferred among institutions (7). This overuse of fractionated regimens may be related to unrealistic concerns about late radiation damage and can expose dying patients to who are not expected to require a re-treatment. On the contrary, the choice of prescribing single doses has the potential to reduce cost and unnecessary visits to the hospital of terminally ill patients. Our high use of single doses for bone metastases in those with shortened survival, indirectly suggests that the fractionation approach was adapted to the end-of-life.

Since the life expectancy of these patients is sometimes too short, when palliative RT is indicated, its impact on quality of life might doubtful. Symptom relief is usually obtained in a wide range of time. When treating painful bone metastases mostly it is achieved at 3–4 weeks (17), while this benefit can be delayed up to months in brain metastases related symptoms (18). Gripp found that half of the patients treated with palliative RT spend most of their remaining time on therapy, of which a large part did not complete the treatment. Out of the patients who died in one month from the first visit, only 16% of survival estimations were correct (2). Despite the fact that the vast majority of our patients completed the treatment, 37 patients did not, of whom 28 died within the first month from the start of the treatment. This means that this small group of patients probably did not benefit from treatment and their life expectancy was expected to be longer.

Predicting survival in terminally patients evaluated for palliative RT is a difficult task since several factors are involved. The clinical predictors’ factor does not seem to be accurate enough to estimate the patient’s real-life expectancy (5, 19). Hemoglobin levels or life-threatening related symptoms, such as dyspnea or cachexia, are also relevant in advanced disease. Hence, it is important to develop survival prediction tools to achieve tailored-end-of-life strategies. In our study, we were able to construct a calculator of 30-DM using the variables with impact on 30-DM in the multivariate analysis. Of them, the ECOG PS is the one with the greatest impact on 30-DM. So that, when a male patient referred for palliative RT presents with an ECOG PS 0–1 and lung cancer, the probability of dying within the first month would be 12.3%, but if the same patient presents with an ECOG PS 2–3, this percentage would increase to 21.4%. Nonetheless, this data is not yet validated. Nowadays, there are several prognostic scores for patients with advanced cancer (9, 19–21), although most of them have not been validated in a prospective cohort of patients treated with palliative RT. Angelo et al. developed a six-parameter decision tree that was able to predict the use of palliative RT in the last 30 days of life. However, it was only applicable to patients with primary lung or bladder cancer (9). A recent study performed by Kain et al. applied the TEACHH model retrospectively to 1,744 consecutive patients. This score consists of six easy-collectable variables specifically addressed to patients referred for palliative RT. They were able to separate patients into three different and clinically relevant survival groups (12). There are few prospective studies using prognostic scores in the palliative RT set. PROGRAD stands out as a prospective study which applies two validated prognostic systems in the initial assessment of patients referred for palliative RT. Using the Palliative Prognostic Index (PPI) and the Number of Risk Factors (NRF) score, they were able to stratify the patients into three groups with different prognoses. PPI score seemed to be the one that best discriminated those patients with the worst prognosis (22).

The relevance of this data is that it can discriminate clinically relevant groups based on scales that are simple to apply and include variables easily collectable in the patient’s first visit. From our part, we have found the presence of factors related to 30-DM that may help develop a more tailored to life expectancy strategy. However, our study is inherently biased by its retrospective design and reflects the clinical practice of a single center, so its interpretation and generalization must be made cautiously. In the current study we only included patients who started the treatment, but a few patients who were planned for palliative RT and died before initiation are not taken into account. For a better understanding of the decision-making, it would be valuable to include for the analysis the patients who were considered unfit for palliative RT. Other variables with demonstrated impact on 30-DM, such as white blood count, dyspnea, or cachexia, could not be collected in a retrospective setting.

RT can provide the necessary relief of symptoms in patients with advanced cancer. However, the use of palliative RT in the last days of life may not be useful. It is therefore important to select appropriately which patients can benefit from palliative RT. While clinical prediction alone seems to be an inaccurate method for decision-making in these patients, 30-DM is objective and can set a clinically relevant time endpoint for symptom relief in patients with short survival. The reliability of survival prediction might be improved with the implementation of objective prognostic systems including variables related to early mortality. Our study provides useful and comparable results with previous, which may be useful to decide whether palliative RT should be indicated or not. In addition, it also may contribute to a better understanding of the patterns of usual clinical practice. There is now a growing body of evidence supporting the implementation of predicting tools in the palliative RT approach. The challenge is to identify those patients who will not benefit from palliative RT in order to provide a better care near the end of life.

## Nomenclature

30-DM, 30-Day Mortality; ECOG, Cooperative Oncology; PS, Performance Status; RT, Radiation Therapy; BS, Better Survival; LS, Lower Survival; PPI, Palliative Prognostic Index; NRF, Number of Risk Factors.

## Data Availability Statement

The raw data supporting the conclusions of this article will be made available by the authors, without undue reservation.

## Ethics Statement

The studies involving human participants were reviewed and approved by the Comitè Ètic d’Investigació Clínica (CEIC) of Vall d’Hebron University Hospital. Written informed consent for participation was not required for this study in accordance with the national legislation and the institutional requirements.

## Author Contributions

Concept and design: MV, MA, and JG. Collection and assembly of data: MV, DM, and AG. Data analysis and interpretation: SP-H and MV. Manuscript writing: MV and JG. Final approval of the manuscript: All authors. All authors contributed to the article and approved the submitted version.

## Conflict of Interest

The authors declare that the research was conducted in the absence of any commercial or financial relationships that could be construed as a potential conflict of interest.
